# Supplementary material for: Twitter (X) use predicts substantial changes in well-being, polarization, sense of belonging, and outrage
Source: Commun Psychol. 2024 Feb 24;2:15. doi: 10.1038/s44271-024-00062-z (PMC11332209; doi:10.1038/s44271-024-00062-z)
Supplement: Supplementary file 1 — Supplementary information [file 44271_2024_62_MOESM1_ESM.pdf]

Supporting Information for:

Twitter (X) use predicts substantial changes in well-being, polarization, sense of belonging, and outrage

Victoria Oldemburgo de Mello[1]* (victoria.mello@mail.utoronto.ca)

Felix Cheung[1] (f.cheung@utoronto.ca)

Michael Inzlicht[12] (michael.inzlicht@rotman.utoronto.ca)

[1] University of Toronto, Toronto, Canada

[2] Rotman School of Management, Toronto, Canada

***Corresponding author:** victoria.mello@mail.utoronto.ca

This file includes:

Supplementary methods
Supplementary note 1
Supplementary note 2
Supplementary note 3
Figures S1-S2
Tables S1-S4
Supplementary references (1-21)

Other supporting materials for this manuscript include the following:

Datasets S1 and S2 (osf.io/e8krz)
R code S1 to S20 (osf.io/e8krz)
Surveys S1 and S2 (osf.io/e8krz)

Supplementary methods

1. Detailed measures and procedures

Data collection took place in four stages: screening for Twitter users, baseline survey, experience sampling surveys, and Twitter data collection. The measurements used in all data analysis are reported in the main manuscript. In this document, we detail all the measures collected in all stages, including the ones not used in any analysis. Full dataset (excluding data with potentially identifiable information) is available at osf.io/e8krz.

Screening Twitter Users

We first set up a study on prolific to screen participants who used Twitter. The screener was set up such that a representative sample of US residents in age, gender, and race would respond to the survey.

In the screener, we first informed participants that they were about to participate in a survey that would evaluate their eligibility for a study. Participants were then asked, "what social media sites do you use?" and they could select multiple options from a list containing "Instagram", "Facebook", "Twitter", "Tiktok", "Youtube", and "Other sites". Multiple platforms were included in the question to avoid socially desirable responding (i.e., responders who answer "yes" to whether they use Twitter just to be eligible for the study).

We then proceeded to ask participants if their Twitter account was private or public, and in case it was private, if they would be willing to make it public for a study. We also asked how much time per week they usually spent on Twitter (the options were "I don't usually spend time on Twitter", "Less than an hour per week", "1-2 hours per week", "More than 3 hours per week"). If participants answered that they did not usually spend time on Twitter, they were automatically redirected to the end of the survey.

We then asked participants about their frequency of Twitter use. They could select one of the options: “less than once a week”, “once a week”, “2-4 times a week”, “5-6 times week”, “once a day”, “more than once a day”. If participants answered “less than once week” they were automatically directed to the end of the study. We then asked participants why they used Twitter, and they could choose among “To connect with friends.”, “To follow the news.”, “To learn new things.”, “To see interesting content.”, “To keep up with content about politics.”, “To see amusing/humorous content.”, “To share my opinions.”, “To see what other people are doing.”. We did not screen participants based on this question.

Finally, we asked participants if they usually have access to a smartphone throughout the day (because the surveys would be sent via text message) and if they would be willing to answer 35 short surveys in a week to earn up to $25. If they answered “yes” to both questions, we then presented them with a short version of the consent form. If they consented, we then asked them for their prolific id (to invite them to the main study) and Twitter handle (for verification).

Baseline Survey

In the baseline survey, we included several questions and scales that we collected data but did not analyze:

Satisfaction with Life. The 5-item instrument by (1) contains items such as “The conditions of my life are excellent” and “I am satisfied with my life”. The items were answered in a 7-point Likert scale ranging from “strongly disagree” to “strongly agree”.

Social Media Use. We asked a comprehensive set of questions about internet use and social media behavior: which devices they used for social media (smartphones, computers, tablets, or others), which social media sites they used from a list containing the most popular sites, whether they felt like they belonged to any online community (and if affirmative, which community),

what types of accounts they followed (e.g.: family members, influencers, businesses, etc). Participants were also instructed to consult their smartphones and report the amount of time per day they spent on social media (e.g., iPhone users were instructed to look at the “screen time” tab under settings). If their device did not offer an estimate of the total time spent on social media, participants were asked to give an estimate.

Loneliness. Loneliness is a measure of well-being. De Jong Gierveld Loneliness Scale measures emotional loneliness (the negative feelings associated with missing intimate relationships) and social loneliness (the experience of lack of belonging in a community). The scale contains 11 items such as “There are people I feel close enough to”. The scale is answered in a 4-Point Likert scale ranging from “definitely not” to “definitely yes" (2).

Sense of Community Online. We also examined contextual sense of community—sense of community on the internet and in internet communities. Our scale was adapted from the sense of belonging in sport instrument (3) to an 8-item scale on feelings about online environments and communities. The scale contains items such as “Other people in this online community take my opinions seriously” and “I have a good bond with others in this community”. Participants answered on a 7-point Likert scale according to how much they agree with each statement, anchors ranging from “strongly agree” to “strongly disagree”.

Social Media Addiction. To measure social media addiction, we adapted The Bergen Facebook Addiction Scale (4). We adapted the items so that they would reflect social media use more generally instead of Facebook use. For instance, the item “How often during the last year have you spent a lot of time thinking about Facebook or planned use of Facebook?” became “How often during the last year have you spent a lot of time thinking about social media or planned use

of social media?” The 6-item scale was answered on a 5-point Likert scale with anchors ranging from “never” to “very often”.

Affective Polarization. Because we were interested in individual-level polarization, we measured affective polarization, “which is the degree to which political partisans dislike, distrust, and avoid the other side” (5). We employed a commonly used measure of affective polarization, the thermometer scale, in which we asked participants to indicate on a 100-point scale how cold (0) to warm (100) they feel about their own party and their opposing party (6).

Political Engagement. With binary questions, we asked participants if they can vote where they reside, if they voted in the last elections, if they contributed to any political campaign in the last elections, if they have participated in any protest in the past year, and if they have demonstrated support for any candidate on social media within the last year.

Ideological Identity. Participants indicated how liberal or conservative they were on a 7-point Likert scale where 0 was “very liberal”, 3 was “neutral”, and 7 was “very conservative”.

News Consumption behaviors. We asked subjects about their frequency of news consumption, what sources they use for news (e.g.: newspaper, cable tv, social media), and if they have ever shared something that they regretted later.

News suspicion. Participants answered in a 5-point Likert scale how much they agreed with each of the following statements: “I am suspicious of mainstream media.”, “I think mainstream media is biased to one side of the story.”, “Social media news are less biased than mainstream media news.”, “I think mainstream media is run by the interests of a powerful group of people.” Scale anchors ranged from “none at all” to “a great deal”.

Government suspicion. To measure the degree to which participants were suspicious about their government, we asked them “How much of the time do you think you can trust the government

in your country to do what is right?” based on the scale developed by (7). Scale anchors ranged from “none at all” to “a great deal”.

Functions of Social Media Use. We wanted to understand the reasons or motivations for social media use. As there were no measures in the literature, we created our own. The first version of the scale contained 32 items divided into 8 different categories of online behavior: social interaction (e.g.: I use social media to keep in touch with family and/or friends), information seeking (e.g.: I use social media to follow the news), pass-time (e.g.: I use social media to relieve boredom when I have idle time), entertainment (e.g.: I use social media to have fun), relaxation (e.g.: I use social media to distract myself from stressful events), information sharing (e.g.: I use social media to share my opinions, thoughts, or reactions with my network), commercial utility (e.g.: I use social media to promote my business/brand), and social comparison (e.g.: I use social media to see what other people are doing with their lives). Participants expressed the frequency they performed each of those functions on a 7-point Likert scale ranging from “never” to “more than once a day”. We performed a preliminary validation of the scale; a full report can be seen in section 2.

Personality. Participants completed the 44-item version of the Big Five Inventory in a 5-point Likert scale measuring five personality dimensions: extraversion, agreeableness, neuroticism, conscientiousness, and openness (8) The scale anchors ranged from “strongly disagree” to “strongly agree”.

2. Preliminary validation of the functions of use scale

In the literature about the psychological effects of social media use, most research considers general measures of social media use. Despite being useful, these measures overlook the potential variability in how people use social media and how different types of use can lead to diverse outcomes.

To overcome this limitation, researchers started to classify social media use based on different patterns of usage. One of the most common classifications considered active (e.g.: posting, commenting, interacting) and passive (e.g.: doom-scrolling, profile watching) uses. The first few studies using this distinction showed consistent associations between passive social media use and decreases in well-being (9, 10). However, a recent meta-analysis failed to find consistent associations between passive use and mental poor health across studies, likely due to the variability in how researchers operationalized the constructs (11). Thus, a promising approach could be to break social media into even more specific behaviors—such as posting, messaging, doomscrolling, etc.

An important distinction is that one social media *behavior* can serve many different *functions*. For instance, doomscrolling can be done to avoid boredom or to look for news; sharing content can be done for endorsement or criticism. Thus, it is important to understand not only the behaviors themselves, but **why** they are done. No studies that investigate the relationship between why people use social media and its psychological outcomes were found in the literature. Hence, we ran a pilot study to develop a measure that captured the functional aspect of social media use—the reasons or motivations for using social media.

The aim of the pilot study was to develop the "functions of social media use" scale. Based on exploratory work on why people use social media (12, 13) we created an initial pool of

32 items divided into potentially 8 different categories (Table S1). Each category corresponded to one function/type of social media use. We tested this initial pool of items in a representative sample of the American population to explore the factor structure of this initial measure.

We ran a Principal Component Analysis on R 4.0.5 (14) to see how many factors would emerge. Based on the eigenvalues, we selected 5 factors. We then ran an exploratory factor analysis using Maximum likelihood extraction method and oblimin rotation on on R 4.0.5 (14) to examine the factor structure. After selecting the items with factor loadings larger than 0.6 or with factor loadings larger than 0.5 with no cross-loadings, we kept and renamed 5 factors: entertainment (e.g.: I use social media to have fun), escapism (e.g.: I use social media to forget about my problems), sharing and interacting (e.g.: I use social media to interact with a community of like-minded people), self-promotion (e.g.: I use social media to promote myself), and information seeking (e.g.: I use social media to self-educate or learn new things). We then ran a Confirmatory Factor Analysis with the lavaan package ((15)to evaluate the five-factor structure. The data fit the model decently, SRMR = .052, RMSEA = .06, CFI = .96, AIC = 13967.83, BIC = 14115.31.

The final scale structure with factor loadings and variance explained are reported in Table S2. The first factor (entertainment) explained 15.88% of the variance. The last factor (information seeking) explained 10.58% of the variance. In total, all five factors explained 61% of the variance.

We explored some relationships between the functions of social media use scale and other relevant measures. We ran a correlation table between the functions of social media use and the personality traits and corrected for false discovery rate on R 4.0.5 (14).The scale was related to personality in predictable ways. For instance, the factor “sharing and interacting” was

positively related to both trait agreeableness, $r(294) = .18$, $p < .00$, and trait extraversion, $r(294) = .30$, $p < .001$. “Escapism” was positively related to neuroticism, $r(294) = .33$, $p < .001$, and negatively related to conscientiousness, $r(294) = -.19$, $p < .05$. Finally, “information seeking” was positively related to trait openness, $r(294) = .17$, $p <.05$. See figure S1 for all relevant relationships.

We also tested for associations between satisfaction with life and several measures of social media use, including the functions of social media use. Total time spent on social media was not significantly related to satisfaction with life, $r(233) = -.06$, $p = .38$. After correcting for multiple testing with false discovery rate, only using social media for escapism was significantly and negatively related to well-being, $r(294) = -.16$, $p = .01$.

Supplementary note 1

Full report of personality interactions. Because the effects of Twitter use on the outcome variables varied a lot across participants, we examined whether any dispositional variables, such as personality, age, and gender, would moderate the relationships. This analysis is important because some researchers suggest that the effect of social media use might be moderated by individual differences (16).

We thus tested for interactions between Twitter use and individual traits. All the interactions are reported in Table S3. Effect sizes R were computed as the square root of the difference in R^2 between a model with and a model without the interaction. Note that all credibility intervals included zero, so none of the interaction models provided conclusive evidence for the interaction effects. This suggests that individual differences might not play such an important role in predicting the effects of social media use. One important limitation to be noted is that our sample size (and thus statistical power) was not planned to detect interactions. To deal with this limitation, we did model comparisons between full (with the interactions) and empty (without the interactions) models by calculating the differences in WAIC. WAIC is a relative measure, so we considered differences in WAIC larger than two Standard Errors as support for one of the models, where positive estimates mean that the empty model was a better fit and negative estimates mean that the full model was a better fit. None of the model comparisons supported the idea that a model with the interaction was a better fit. If anything, the empty model was a better fit in two of the 28 comparisons (polarization predicted by the interaction between Twitter use and agreeableness and Twitter use and openness).

Supplementary note 2

Further Exploratory Analyses. In this section, we report some exploratory analyses.

4.1 The relationship between functions and behaviors of use

To better understand the meaning for these different factors in the functions of use scale, we created a mutual information table from the frequencies of each behavior and function in Table S6. The function and behavior that co-occurred most often was using Twitter for entertainment and scrolling down the feed (819 co-occurrences in total) and the least common co-occurrence was using Twitter for self-promotion and checking the trending topics (2 occurrences).

4.2 The relationship between outrage and polarization

We wanted to know if experiences of outrage would be related to changes in polarization. Because expressions of outrage tend to be bound within groups (17), and Twitter groups tend to be highly segregated (18), we expected that higher outrage would lead to higher levels of polarization. To test our hypothesis, we ran Bayesian multilevel models to test whether experiences of outrage would predict polarization, while controlling for past levels of polarization and outrage. However, outrage was not related to polarization, even if Twitter use predicted increases in both. This finding suggests that the mechanisms for both psychological processes might have different and unrelated causes on social media—outrage limited to the momentary emotional experience of encountering a moral violation (19) and polarization related to long-lasting affective and cognitive processes (20).

For the models that included an interaction, we also estimated model fit and compared it to a model without the interaction. We centered the predictors around the participant mean for level 1 variables and calculated the participant mean for level 2 variables. To test model fit, we

calculate the difference in Watanabe-Akaike information criterion (WAIC) between a model without the interaction and a model with the interaction using the function *compare_ic* from the brms package (21).We then report ΔWAIC, the difference in WAIC between the two models and the estimated standard error of that difference (SE). We considered differences in WAIC larger than two SEs as evidence that one of the models has better model fit.

Table S3 breaks down the interactions between personality, twitter use, and the main outcome variables. No results excluded zero and no models with interactions had better model fit than the model without the interactions.

4.3 The moderating role of political identity

We estimated the interaction between Twitter use and well-being, outrage, and affective polarization. None of these interactions had substantial results, $b_{well-being}$ = .07, CrI = [-.02, .16]; $b_{outrage}$ = -.07, CrI = [-.15, .01]; $b_{polarization}$= .02, CrI = [-.01, .04]. This result could have resulted from a lack of statistical power to detect those effects. Because our sample had few conservatives (34 out of 252 participants), it is possible that we do not have enough observations to infer about the role of ideology in the relationship between Twitter use and these outcome variables.

Supplementary note 3

5.1. Behaviors and functions of Twitter Use prevalence

Some readers might be interested in the prevalence of Twitter use behaviors and Twitter use functions across participants. We have made the entire dataset available at osf.io/e8krz.

5.2. Compliance Information

Participants have filled out, on average, 24.67 surveys out of the 35 sent (calculated across participants), with an SD of 7.15. The median of responses across participants was 26, and the mode was both 31 and 32 (17 observations each). The lowest number of surveys that a participant could answer and still be included in our analysis was 9 (the actual sample minimum). 11 participants answered all 35 surveys.

Supplementary references

1. E. Diener, R. A. Emmons, R. J. Larsem, S. Griffin, The Satisfaction With Life Scale. *J Pers Assess* **49**, 71–75 (1985).
2. J. de Jong-Gierveld, F. Kamphuls, The Development of a Rasch-Type Loneliness Scale: *http://dx.doi.org/10.1177/014662168500900307* **9**, 289–299 (2016).
3. J. B. Allen, The perceived belonging in sport scale: Examining validity. *Psychol Sport Exerc* **7**, 387–405 (2006).
4. C. Schou Andreassen, T. Torsheim, G. Scott Brunborg, S. Pallesen, Development of a Facebook Addiction Scale. *Psychol Rep* **110**, 501–517 (2012).
5. A. E. Wilson, V. Parker, M. Feinberg, Polarization in the contemporary political and media landscape. *Curr Opin Behav Sci* **34**, 223–228 (2020).
6. S. Iyengar, Y. Lelkes, M. Levendusky, N. Malhotra, S. J. Westwood, The Origins and Consequences of Affective Polarization in the United States. *Annu. Rev. Polit. Sci* **22**, 129–146 (2019).
7. J. Gershtenson, D. L. Plane, Trust in Government 2006 American National Election Studies Pilot Report (2007) (August 9, 2021).
8. O. P. John, S. Srivastava, "The Big-Five Trait Taxonomy: History, Measurement, and Theoretical Perspectives" in Handbook of personality: Theory and research, L. Pervin and O.P. John (2nd ed.). New York: Guilford.
9. P. Verduyn, *et al.*, Passive facebook usage undermines affective well-being: Experimental and longitudinal evidence. *J Exp Psychol Gen* **144**, 480–488 (2015).
10. P. Verduyn, O. Ybarra, M. Résibois, J. Jonides, E. Kross, Do Social Network Sites Enhance or Undermine Subjective Well-Being? A Critical Review. *Soc Issues Policy Rev* **11**, 274–302 (2017).
11. P. M. Valkenburg, I. I. van Driel, I. Beyens, The Associations of Active and Passive Social Media Use With Well-being: A Critical Scoping Review https:/doi.org/10.31234/OSF.IO/J6XQZ (February 24, 2022).
12. G. M. Chen, Tweet this: A uses and gratifications perspective on how active Twitter use gratifies a need to connect with others. *Comput Human Behav* **27**, 755–762 (2011).
13. A. Whiting, D. Williams, Why people use social media: a uses and gratifications approach (2013) https:/doi.org/10.1108/QMR-06-2013-0041 (November 22, 2020).
14. R Core Team, R core team (2021). *R: A language and environment for statistical computing. R Foundation for Statistical Computing, Vienna, Austria. URL http://www. R-project. org* (2021).
15. Y. Rosseel, lavaan: An R Package for Structural Equation Modeling. *J Stat Softw* **48**, 1–36 (2012).
16. E. Kross, *et al.*, Social Media and Well-Being: Pitfalls, Progress, and Next Steps. *Trends Cogn Sci* (2020) https:/doi.org/10.1016/j.tics.2020.10.005 (November 11, 2020).

17. W. J. Brady, *et al.*, Emotion shapes the diffusion of moralized content in social networks. *Proc Natl Acad Sci U S A* **114**, 7313–7318 (2017).
18. P. Barberá, J. T. Jost, J. Nagler, J. A. Tucker, R. Bonneau, Tweeting From Left to Right: Is Online Political Communication More Than an Echo Chamber? *Psychol Sci* **26**, 1531–1542 (2015).
19. Y. R. Avramova, Y. Inbar, Emotion and moral judgment. *WIREs Cogn Sci* **4**, 169–178 (2013).
20. S. Iyengar, G. Sood, Y. Lelkes, Affect, not ideology: A social identity perspective on polarization. *Public Opin Q* **76**, 405–431 (2012).
21. P.-C. Bürkner, brms: An R Package for Bayesian Multilevel Models using Stan (June 21, 2022).

Figures

Figure S1

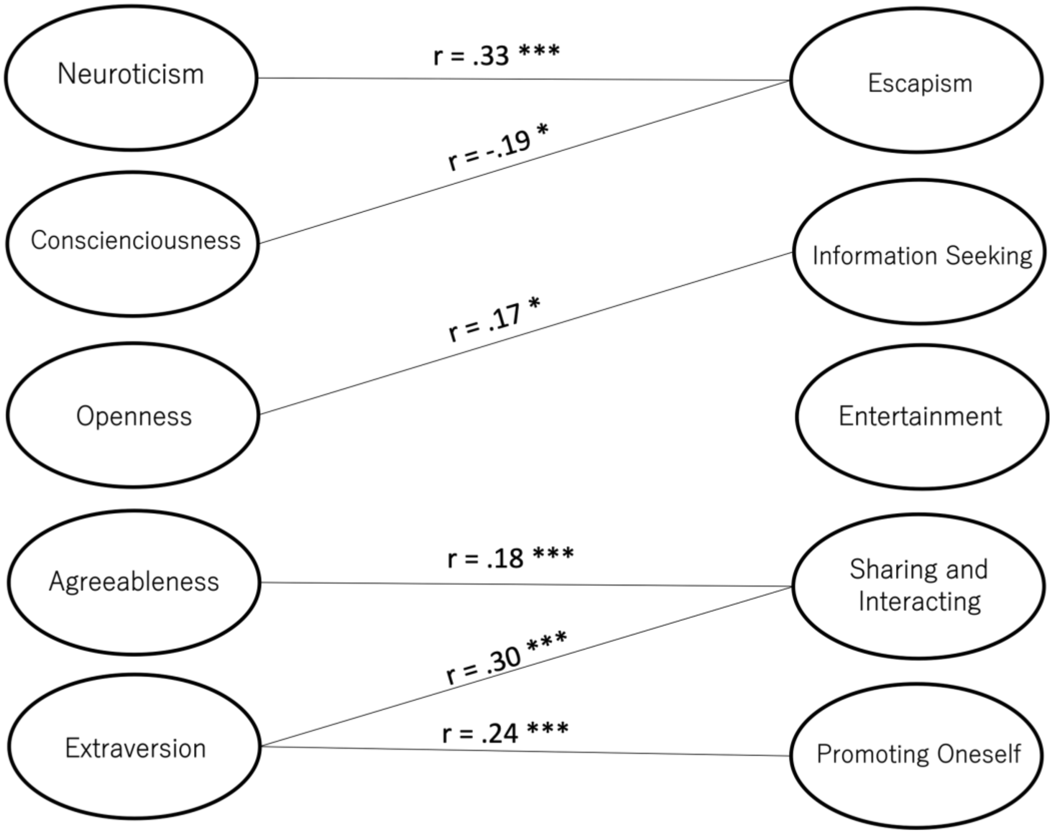

Figure S1. Correlations between functions of use and personality factors. Shapes on the left side of the image represent personality factors, shapes on the right side represent the functions of use. (*) indicates significance level at p < .05, (***) indicates significance level at p <.001.

Figure S2.

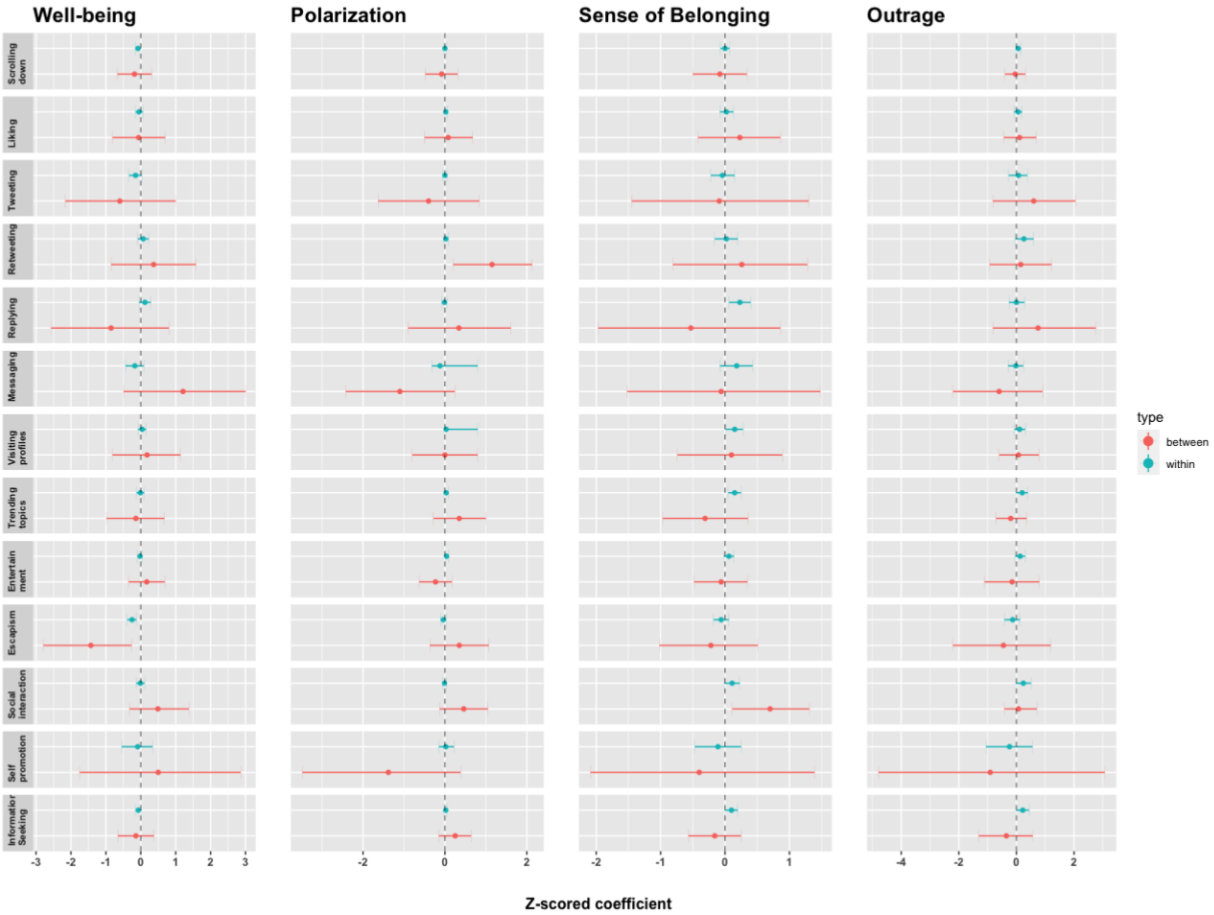

Figure S2. Forest Plot of All the Effect Sizes for Twitter Behaviors and Functions. Rows 1-8 represent behaviors, rows 9-13 represent functions of use. Each column represents an outcome variable. In each plot, blue points represent the relationship point estimate at the within participants level of analysis, while pink points represent the relationship point estimate at the between participants level of analysis. Whiskers represent credibility intervals.

Tables

Table S1.

**Social Interaction**
Keep in touch with family and/or friends.
Meet people with common interests.
Interacting with members of my social community.
interact with a community of like-minded people.
networking for work or school purposes.
**Information Seeking**
Follow the news.
Self-educate (learn new things).
Find information about businesses and products.
Find information about events and gatherings.
Get inspiration.
**Pass-time**
Pass my time when I do not have much to do.
Relieve boredom when I have idle time.
Relieve boredom during class or work.
**Entertainment**
Have fun.
Enjoy myself.

See fun content.
Entertain myself.
**Relaxation**
Relax.
Distract myself from stressful events.
Forget about my problems.
Take my mind off my regular life.
**Information sharing**
Share my opinions, thoughts, or reactions with my network.
Share personal events that happened to me with my network.
Share my work with my network.
Show my network that I have some characteristic or virtue.
**Commercial**
To promote my business/brand.
To promote myself.
Monetize (e.g.: get paid for posting).
Online shopping.
**Knowledge about others/Social comparison**
To see what other people are doing with their lives.
To compare myself with other people online.
To see how I am doing relative to other people.

Table S1. *Initial Items in the "Functions of Social Media Use" Scale*.

Table S2. *Final Functions of Use Scale with factor loadings and variance explained for each factor.*

Factor	Item	Loadings	Variance Explained
Factor 1 - Entertainment	...enjoy myself.	0.818	15.88%
	...relax.	0.748	
	...have fun.	0.732	
Factor 2 - Escapism	...forget about my problems	0.880	13.96%
	...take my mind off my regular life.	0.860	
	...distract myself from stressful events.	0.669	
Factor 3 - Sharing and Interacting	...interact with a community of like-minded people.	0.732	11.31%
	...meet people with common interests.	0.616	
	...share my opinions, thoughts, or reactions with my network.	0.585	
Factor 4 - Promoting oneself	...promote myself.	0.880	9.24%
	...promote my business/brand.	0.821	
	...monetize (e.g.: get paid for posting).	0.613	
Factor 5 - Information Seeking	...get inspiration.	0.744	10.58%
	...self-educate (learn new things).	0.691	

...find information about businesses and products.	0.645

Table S3. Main Effects of Twitter Use on Outcome Variables.

OUTCOME	TWITTER USE (WITHIN)	TWITTER USE (BETWEEN)
**BENCHMARK** (WELL-BEING ~ SOCIAL INTERACTION)	b_{within} = .15, 95% CrI = [.02, .28]	$b_{between}$ = .27, 95% CrI = [-.02, .56]
**WELL-BEING**	**b_{within} = -0.10, 95% CrI = [-.15, -.04]**	$b_{between}$ = -0.09, 95% CrI = [-.42, .25]
**BOREDOM**	**b_{within} = .22, 95% CrI = [.14, .30]**	**$b_{between}$ = .50, 95% CrI = [.18, .83]**
**LONELINESS**	b_{within} = .05, 95% CrI [-.01, .11]	**$b_{between}$ = .42, 95% CrI = [.05, .78]**
**ANXIETY**	b_{within} = .02, 95% CrI = [-.03, .08]	$b_{between}$ = .16, 95% CrI = [-.18, .50]
**SENSE OF BELONGING**	**b_{within} =.11, 95% CrI = [.04, .17]**	$b_{between}$= .01, 95% CrI = [-.28, .30]
**POLARIZATION**	**b_{within} = .03, 95% CrI = [.00, .06]**	$b_{between}$= .20, 95% CrI = [-.05, .46]
**OUTRAGE**	**b_{within}= .19, 95% CrI = [.10, .28]**	$b_{between}$ = .15, 95% CrI = [-.12, .42]

Table S3. Main effects of Twitter use on the outcome variables. Twitter use (within) is the coefficient for Twitter use at the within-subjects level, while Twitter use (between) is the between-subjects level. The first row (in dark grey) is the result of the positive control condition, an effect that would be expected in this sample.

Table S4. Effects for behaviors of use/functions of use on main outcome variables

	Predictor	Well-being	Polarization	Sense Community	Outrage
**Behaviors**	Retweeting	bb =0.36, CrI = [-0.86, 1.58]	**bb =1.16, CrI = [0.21, 2.15]**	bb =0.26, CrI = [-0.81, 1.28]	bb =0.2, CrI = [-0.87, 1.26]
	Retweeting	bw =0.07, CrI = [-0.09, 0.23]	bw =0.03, CrI = [-0.04, 0.09]	bw =0.02, CrI = [-0.16, 0.2]	bw =0.28, CrI = [-0.04, 0.6]
	Liking	bb =-0.06, CrI = [-0.8, 0.69]	bb =0.06, CrI = [-0.5, 0.65]	bb =0.23, CrI = [-0.42, 0.86]	bb =0.11, CrI = [-0.48, 0.67]
	Liking	bw =-0.05, CrI = [-0.15, 0.06]	bw =0.03, CrI = [-0.01, 0.07]	bw =0.02, CrI = [-0.08, 0.13]	bw =0.07, CrI = [-0.08, 0.21]
	Tweeting	bb =-0.58, CrI = [-2.19, 1.06]	bb =-0.45, CrI = [-1.7, 0.77]	bb =-0.09, CrI = [-1.45, 1.3]	bb =0.56, CrI = [-0.81, 1.96]
	Tweeting	bw =-0.15, CrI = [-0.34, 0.03]	bw =-0.01, CrI = [-0.06, 0.05]	bw =-0.04, CrI = [-0.22, 0.15]	bw =0.06, CrI = [-0.26, 0.37]
	Commenting	bb =-0.86, CrI = [-2.59, 0.77]	bb =0.36, CrI = [-0.91, 1.62]	bb =-0.53, CrI = [-1.97, 0.86]	bb =0.67, CrI = [-0.83, 2.41]
	Commenting	bw =0.11, CrI = [-0.06, 0.29]	bw =-0.02, CrI = [-0.08, 0.05]	**bw =0.23, CrI = [0.06, 0.4]**	bw =0.01, CrI = [-0.27, 0.28]
	Messaging	bb =1.23, CrI = [-0.43, 2.96]	bb =-1.13, CrI = [-2.42, 0.19]	bb =-0.06, CrI = [-1.52, 1.48]	bb =-0.56, CrI = [-2.13, 0.91]
	Messaging	bw =-0.18, CrI = [-0.44, 0.1]	bw =-0.12, CrI = [-0.33, 0.08]	bw =0.18, CrI = [-0.08, 0.43]	bw =-0.02, CrI = [-0.29, 0.25]
	Visiting	bb =0.17, CrI = [-0.83, 1.14]	bb =0.02, CrI = [-0.74, 0.77]	bb =0.1, CrI = [-0.74, 0.89]	bb =0.05, CrI = [-0.63, 0.74]
	Visiting	bw =0.04, CrI = [-0.08, 0.15]	bw =0.04, CrI = [-0.01, 0.08]	**bw =0.15, CrI = [0.01, 0.28]**	bw =0.11, CrI = [-0.07, 0.3]

	Trending Topics	bb =-0.14, CrI = [-0.96, 0.67]	bb =0.32, CrI = [-0.32, 0.95]	bb =-0.31, CrI = [-0.97, 0.36]	bb =-0.19, CrI = [-0.72, 0.35]
	Trending Topics	bw =-0.01, CrI = [-0.12, 0.09]	bw =0.03, CrI = [-0.03, 0.09]	**bw =0.15, CrI = [0.05, 0.25]**	bw =0.2, CrI = [0, 0.41]
	Scrolling	bb =-0.18, CrI = [-0.67, 0.31]	bb =-0.09, CrI = [-0.47, 0.3]	bb =-0.08, CrI = [-0.5, 0.34]	bb =-0.05, CrI = [-0.41, 0.31]
	Scrolling	**bw =-0.08, CrI = [-0.15, -0.01]**	bw =0.01, CrI = [-0.02, 0.03]	bw =0, CrI = [-0.07, 0.07]	bw =0.07, CrI = [-0.02, 0.16]
**Functions**	Escapism	**bb =-1.4, CrI = [-2.78, -0.25]**	bb =0.35, CrI = [-0.37, 1.07]	bb =-0.22, CrI = [-1.02, 0.51]	**bb =1.72, CrI = [0.62, 2.9]**
	Escapism	**bw =-0.25, CrI = [-0.39, -0.12]**	bw =-0.04, CrI = [-0.1, 0.03]	bw =-0.06, CrI = [-0.18, 0.06]	**bw =0.33, CrI = [0.11, 0.55]**
	Entertainment	bb =0.16, CrI = [-0.35, 0.66]	bb =-0.23, CrI = [-0.63, 0.17]	bb =-0.06, CrI = [-0.48, 0.35]	bb =-0.33, CrI = [-0.67, 0]
	Entertainment	bw =-0.02, CrI = [-0.09, 0.05]	**bw =0.04, CrI = [0.01, 0.08]**	bw =0.06, CrI = [-0.02, 0.14]	bw =0.04, CrI = [-0.05, 0.12]
	Self-promotion	bb =0.5, CrI = [-1.78, 2.86]	bb =-1.38, CrI = [-3.48, 0.38]	bb =-0.4, CrI = [-2.09, 1.39]	bb =2.96, CrI = [-3.41, 10.14]
	Self-promotion	bw =-0.09, CrI = [-0.55, 0.35]	bw =0.02, CrI = [-0.15, 0.22]	bw =-0.11, CrI = [-0.47, 0.25]	bw =0.23, CrI = [-0.4, 0.78]
	Information Seeking	bb =-0.13, CrI = [-0.66, 0.39]	bb =0.25, CrI = [-0.15, 0.64]	bb =-0.16, CrI = [-0.57, 0.25]	bb =0.3, CrI = [-0.04, 0.67]
	Information Seeking	bw =-0.07, CrI = [-0.15, 0.01]	bw =0.02, CrI = [-0.02, 0.05]	bw =0.1, CrI = [0, 0.2]	**bw =0.14, CrI = [0.03, 0.25]**
	Social Interaction	bb =0.47, CrI = [-0.33, 1.36]	bb =0.46, CrI = [-0.13, 1.05]	**bb =0.7, CrI = [0.11, 1.31]**	bb =0.14, CrI = [-0.43, 0.7]

	Social Interaction	bw =-0.01, CrI = [-0.13, 0.11]	bw =-0.01, CrI = [-0.06, 0.03]	bw =0.11, CrI = [-0.01, 0.23]	bw =0.1, CrI = [-0.12, 0.32]

Table S4. Main effects of twitter behaviors and twitter functions on well-being, polarization, and well-being. Effects for behaviors of use/functions of use on main outcome variables. b_w is the effect within-person (how much variability explained within each individual between each time point), and b_b is the effect between-person (how much variability explained between individual differences). b is the estimate and CrI is the 95% credibility interval (lower and upper), respectively. All models were run controlling for past levels of the DV and the IV. Results in bold that the credibility interval did not include zero.
