# Supplementary material for: Twitter (X) use predicts substantial changes in well-being, polarization, sense of belonging, and outrage
Source: Commun Psychol. 2024 Feb 24;2:15. doi: 10.1038/s44271-024-00062-z (PMC11332209; doi:10.1038/s44271-024-00062-z)
Supplement: Supplementary file 3 — Reporing Summary [file 44271_2024_62_MOESM3_ESM.pdf]

nature portfolio

Corresponding author(s): *Double-blind peer review submissions: write DBPR and your manuscript number here instead of author names.*

Last updated by author(s): *YYYY-MM-DD*

Reporting Summary

Nature Portfolio wishes to improve the reproducibility of the work that we publish. This form provides structure for consistency and transparency in reporting. For further information on Nature Portfolio policies, see our Editorial Policies and the Editorial Policy Checklist.

Statistics

For all statistical analyses, confirm that the following items are present in the figure legend, table legend, main text, or Methods section.

n/a	Confirmed	
☐	☒	The exact sample size (*n*) for each experimental group/condition, given as a discrete number and unit of measurement
☐	☒	A statement on whether measurements were taken from distinct samples or whether the same sample was measured repeatedly
☐	☒	The statistical test(s) used AND whether they are one- or two-sided<br>*Only common tests should be described solely by name; describe more complex techniques in the Methods section.*
☐	☒	A description of all covariates tested
☐	☒	A description of any assumptions or corrections, such as tests of normality and adjustment for multiple comparisons
☐	☒	A full description of the statistical parameters including central tendency (e.g. means) or other basic estimates (e.g. regression coefficient) AND variation (e.g. standard deviation) or associated estimates of uncertainty (e.g. confidence intervals)
☐	☒	For null hypothesis testing, the test statistic (e.g. F, t, r) with confidence intervals, effect sizes, degrees of freedom and P value noted<br>*Give P values as exact values whenever suitable.*
☐	☒	For Bayesian analysis, information on the choice of priors and Markov chain Monte Carlo settings
☐	☒	For hierarchical and complex designs, identification of the appropriate level for tests and full reporting of outcomes
☐	☒	Estimates of effect sizes (e.g. Cohen's *d*, Pearson's *r*), indicating how they were calculated

Our web collection on statistics for biologists contains articles on many of the points above.

Software and code

Policy information about availability of computer code

Data collection	Qualtrics survey, SurveySignal Distribution.
Data analysis	R, brms package for R.

For manuscripts utilizing custom algorithms or software that are central to the research but not yet described in published literature, software must be made available to editors and reviewers. We strongly encourage code deposition in a community repository (e.g. GitHub). See the Nature Portfolio guidelines for submitting code & software for further information.

Data

Policy information about availability of data

All manuscripts must include a data availability statement. This statement should provide the following information, where applicable:

- Accession codes, unique identifiers, or web links for publicly available datasets
- A description of any restrictions on data availability
- For clinical datasets or third party data, please ensure that the statement adheres to our policy

Data, code, and materials are available at osf.io/e8krz.

Human research participants

Policy information about studies involving human research participants and Sex and Gender in Research.

Reporting on sex and gender	51% self-identified as male, 49% self-identified as female.
Population characteristics	These individuals had an average age of 42.99 years (SD = 14.06), were 51% male, and had a racial composition roughly representative of the US population (72% white, 14% black, 7.5% Asian, 4% mixed race, and 2.5% other ethnicities). 67.5% of the sample self-identified as liberal, 13.5% as conservative, 16.3% as moderates, and 2.8% did not report political ideology. On Twitter, 14% of the users consider themselves to be very conservative and about 64% of heavy users identify as Democrats (compared with 55% of light users) (7).
Recruitment	We aimed to collect data from 300 participants. To get as close as possible to a representative sample of Twitter users in age, gender, and race, we used quota sampling on Prolific Academic (48), where we screened participants for Twitter use, inviting those who used the platform at least twice a week to participate in our study. 404 participants accepted the invitation to join the study and answered the baseline survey. We ran the screening and the invitations to the study in batches (8 in total) until we had at least 300 participants answering at least one of the experience sampling surveys (N=309). Data was collected from March to June 2021.
Ethics oversight	The study was approved by the Human Participant Ethics Protocol Submission at the University of Toronto under the protocol number 40106.

Note that full information on the approval of the study protocol must also be provided in the manuscript.

Field-specific reporting

Please select the one below that is the best fit for your research. If you are not sure, read the appropriate sections before making your selection.

☐ Life sciences ☒ Behavioural & social sciences ☐ Ecological, evolutionary & environmental sciences

For a reference copy of the document with all sections, see nature.com/documents/nr-reporting-summary-flat.pdf

Behavioural & social sciences study design

All studies must disclose on these points even when the disclosure is negative.

Study description	Observational quantitative repeated-measures study.
Research sample	Our final sample was 252 participants. These individuals had an average age of 42.99 years (SD = 14.06), were 51% male, and had a racial composition roughly representative of the US population (72% white, 14% black, 7.5% Asian, 4% mixed race, and 2.5% other ethnicities). 67.5% of the sample self-identified as liberal, 13.5% as conservative, 16.3% as moderates, and 2.8% did not report political ideology. On Twitter, 14% of the users consider themselves to be very conservative and about 64% of heavy users identify as Democrats (compared with 55% of light users). These numbers suggest that our sample was roughly representative of Twitter user in terms of political ideology. While our sample was not strictly representative, it was an improvement over many social media studies that rely on convenience samples of undergraduate students or focus on the most active Twitter users (Pew Research Center, 2021).
Sampling strategy	Using multi-level simulations (64) (with d =.15 and 80% statistical power), we determined that a sample size of 220-300 participants (each providing 20 observations) would suffice. We ultimately aimed to collect data from 300 participants. To get as close as possible to a representative sample of Twitter users in age, gender, and race, we used quota sampling on Prolific Academic (48), where we screened participants for Twitter use, inviting those who used the platform at least twice a week to participate in our study.
Data collection	404 participants accepted the invitation to join the study and answered the baseline survey. We ran the screening and the invitations to the study in batches (8 in total) until we had at least 300 participants answering at least one of the experience sampling surveys (N=309). Participants did not know about the purpose and hypotheses of the study.
Timing	Data was collected from March to June 2021. We had 8 cohorts during this time.
Data exclusions	We excluded participants who failed attention checks in the baseline survey. We have also excluded participants who did not respond to enough surveys (less than 9).
Non-participation	Any participants who failed to provide enough data points (less than 9 out of 35 surveys) were excluded. Participants with few data points made the statistical models impossible to converge.
Randomization	There was no randomization in this study.

Reporting for specific materials, systems and methods

We require information from authors about some types of materials, experimental systems and methods used in many studies. Here, indicate whether each material, system or method listed is relevant to your study. If you are not sure if a list item applies to your research, read the appropriate section before selecting a response.

Materials & experimental systems

n/a	Involved in the study
☒	☐ Antibodies
☒	☐ Eukaryotic cell lines
☒	☐ Palaeontology and archaeology
☒	☐ Animals and other organisms
☒	☐ Clinical data
☒	☐ Dual use research of concern

Methods

n/a	Involved in the study
☒	☐ ChIP-seq
☒	☐ Flow cytometry
☒	☐ MRI-based neuroimaging
